# Association between high-density lipoprotein cholesterol and 7-autoantibodies: a study on physical examination data from 2018 to 2023

**DOI:** 10.3389/fendo.2025.1504266

**Published:** 2025-03-06

**Authors:** Yang Zhou, Yongbing Sun, Qi Qiao, Xin Qi, Xinbei Lin, Yawei Du, Ao Liu, Jing Zhou, Xue Lv, Zhonglin Li, Xiaoling Wu, Zhi Zou, Michael Zhang, Jiadong Zhu, Feifei Shang, Hao Li, Yongli Li

**Affiliations:** ^1^ Department of Medical Imaging, Henan Provincial People’s Hospital, Zhengzhou University People’s Hospital, Zhengzhou, Henan, China; ^2^ Department of Medical Imaging, Henan Provincial People’s Hospital, Xinxiang Medical University, Zhengzhou, Henan, China; ^3^ Department of Medical Imaging, Henan University People’s Hospital, Henan Provincial People’s Hospital, Zhengzhou, Henan, China; ^4^ Henan Provincial Research Center of Clinical Medicine of Nephropathy, Zhengzhou University People’s Hospital, Henan University People’s Hospital, Henan Provincial People’s Hospital, Zhengzhou, Henan, China; ^5^ Department of Health Management, Henan Provincial People’s Hospital, Zhengzhou, Henan, China; ^6^ Department of Nuclear Medicine, Henan Provincial People’s Hospital, Zhengzhou, Henan, China; ^7^ Sevenoaks Health Management Center, Canada-Canada Institute of Health Engineering, University of Manitoba, Winnipeg, MB, Canada; ^8^ Chronic Health Management Laboratory, Department of Health Management, Henan Provincial People’s Hospital, Zhengzhou, Henan, China; ^9^ Department of Health Management, Fuwai Central China Cardiovascular Hospital, Zhengzhou, Henan, China

**Keywords:** HDL-C, 7-AABs, lung cancer, Chinese adults, physical examinations

## Abstract

**Background:**

Limited research has explored the effect of high-density lipoprotein cholesterol (HDL-C) on lung cancer’s seven autoantibodies (7-AABs). This study investigated the association between serum HDL-C and 7-AABs among 5,574 Chinese adults aged ≥ 18 years from January 2018 to December 2023.

**Methods:**

This cross-sectional study utilized physical examination data from the Department of Health Management at Henan Provincial People’s Hospital. The associations between HDL-C and autoantibodies, such as tumor protein 53(P53), SRY-box containing gene 2 (SOX2), and ATP-dependent RNA helicase 4-5 (GBU4-5), were modeled using a restricted cubic spline logistic regression model.

**Results:**

After the adjustment for factors, such as age and body mass index, the binary logistic regression model showed distinct correlations between serum HDL-C levels and autoantibodies, including P53, SOX2, and 7-AABs. Restricted cubic spline logistic regression analysis indicated that the increased level of serum HDL-C was associated with a decreased risk of positive P53 (all participants: HDL-C: 1.227–1.366 mmol/L, *P*_HDL-C_=0.028), SOX2 (all participants: HDL-C ≥ 1.227 mmol/L, *P*_HDL-C_ =0.021; all women: HDL-C ≥ 1.224 mmol/L, *P*_HDL-C_=0.037), GBU4-5 (all women: HDL-C ≥ 1.269 mmol/L, *P*_HDL-C_=0.039), and 7-AABs (all women: HDL-C ≥ 1.224 mmol/L, *P*_HDL-C_=0.015). In women, HDL-C levels between 1.163 and 1.224 mmol/L correlated with an increased risk of positive 7-AABs test results.

**Conclusions:**

Elevated HDL-C levels exhibited an independent association with a reduced risk of positivity for 7-AABs of lung cancer, especially in the female physical examination population. These findings suggest that high HDL-C levels may play a role in hindering lung cancer development with gender differences. However, further confirmation is still needed in the future.

## 1 Introduction

Lung cancer is a prevalent and important cause of mortality worldwide (1, 2). The incidence of lung cancer has been rapidly increasing, and 3.6 million cases are predicted for 2040 (3). In China, lung cancer is the most common malignant tumor in males and has the highest mortality rate in men and women. This condition poses a serious health threat to the population (4). During lung cancer development, tumor tissues produce lung cancer-related proteins, which stimulate the immune system to produce highly specific autoantibodies. These autoantibodies can serve as biomarkers for lung cancer (5). In a large-scale clinical study focusing on the Chinese population, Ren et al. demonstrated the clinical utility of a set of autoantibodies known as seven autoantibodies (7-AABs) for early lung cancer screening (6). These autoantibodies include tumor protein 53 (p53), G antigen 7 (GAGE7), protein-encoding gene product 9.5 (PGP9.5), cancer/testis-associated antigen (CAGE), melanoma antigen A1 (MAGEA1), SRY-box containing gene 2 (SOX2), and ATP-dependent RNA helicase 4-5 (GBU4-5). Several studies have reported a diagnostic specificity for lung cancer as high as 90% (7). The China Food and Drug Administration has recognized the 7-AABs spectrum as the sole autoantibody spectrum for early lung cancer screening and diagnosis. Numerous studies have explored the correlation between metabolic syndrome (MS) and lung and other cancers (8, 9). High-density lipoprotein cholesterol (HDL-C), a component of MS (10), also correlates with the progression of various cancers (11, 12). Crudele et al. observed that low HDL levels can be used as a novel marker to predict hepatocellular carcinoma development in patients with liver fibrosis (13). ŞAHIN et al. reported that HDL levels were lower in the lung cancer metastasis group compared with the healthy group (14). A retrospective observational study conducted by Luo et al. revealed that high HDL-C levels in lung cancer patients prior to chemotherapy were independent predictors of a long disease-free survival. This condition suggests that monitoring HDL-C fluctuations can be a valuable predictor for lung cancer and blood lipid levels should be monitored long-term for effective tumor management (15). Lin et al. confirmed a significant inverse association between HDL-C levels and the risk of lung carcinoma (16). HDL-C may regulate cancer development by influencing proliferative and inflammatory pathways, which leads to a favorable anticancer state (17). HDL-C is closely related to lung cancer progression, which can be aid in identifying and monitoring high-risk patients (18). Elevated HDL levels can reduce oxidative stress and proinflammatory molecule levels in cancer cells and the tumor microenvironment (TME) (19).

However, no research explored the association between 7-AABs and serum HDL-C levels among the Chinese population. The effect of fluctuations in serum HDL-C levels on the screening results of 7-AABs should be explored to provide new ideas for their joint detection to improve the efficiency of lung cancer screening and realize the early detection and diagnosis of lung cancer in clinical practice. Therefore, in this study, we assumed that an association exists between 7-AABs and HDL-C levels. The homogeneous sample in this study aided in elucidating the association between HDL-C and lung cancer autoantibodies among Chinese adults and the findings may provide guidance for the use of HDL-C as an early screening indicator for lung cancer, which may compensate for the shortcomings of the clinical lung cancer screening process.

This research aimed to reveal the potential correlations between HDL-C and 7-AABs and analyze epidemiological data to elucidate such association. By investigating the influence of serum HDL-C on the detection results for 7-AABs, this study sought to provide new insights and evidence for the anticancer effects and lung cancer screening effects of HDL-C.

## 2 Methods

### 2.1 Study subjects

**Figure 1:**
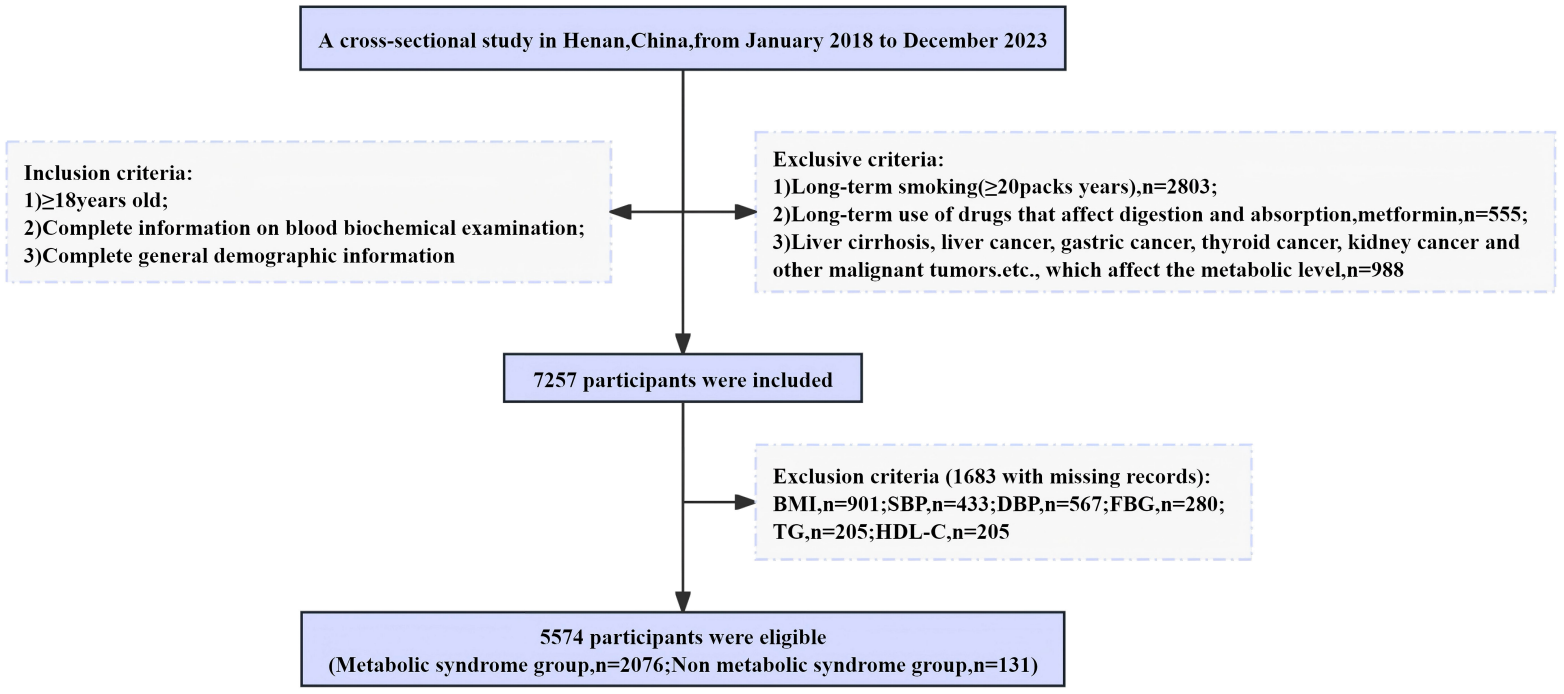
Flowchart of participants selection.

Data were obtained from the physical examination database of the medical examination center at Henan Provincial People’s Hospital from 2018 to 2023. The inclusion criteria for this study comprised the following: (1) adult medical examination personnel over 18 years of age, (2) complete blood biochemical examination information, and (3) complete general demographic information. The exclusion criteria included the following: (1) a long-term smoking history of 30 pack years (20, 21); (2) long-term use of lipid-lowering drugs; (3) a history of metabolic disorder diseases, endocrine diseases, or various malignant tumors. Trained personnel conducted face-to-face interviews for the collection of basic data, including age, sex, metabolic indicators, medical history, and medication history. Initially, 7,257 participants were included, but 1,683 were subsequently excluded due to incomplete blood biochemical index data. Ultimately, the study incorporated 5,574 participants (**Figure 1**).

### 2.2 Methods of research

Before the survey, all study participants underwent uniform training to ensure the accuracy and reliability of data acquisition. The participants’ underlying data were collected through a standardized questionnaire, which included information on their medical history related to malignant tumors, liver and kidney diseases, thyroid disorders, and the intake of lipid metabolism-regulating drugs. After the participants have completed the survey questionnaires, the data were collated, checked, and verified.

### 2.3 Physical examination

① After a 12 h fasting period, professional medical staff measured the height, weight, and blood pressure of the participants, who were wearing light clothing and had removed their shoes, in the morning. Each measurement was performed twice and averaged to minimize errors. ② Body mass index (BMI) was calculated as weight divided by height squared (kg/m^2^).

### 2.4 Laboratory measurements

#### 2.4.1 Fasting blood glucose and lipid measurements

① FBG detection: All subjects fasted for 8 h or more before fasting venous blood samples were collected to avoid heavy drinking and eating. ② Blood lipid tests: A total of 5 mL venous blood was obtained from the participants and sent to the laboratory for analysis of triglyceride (TG) and HDL-C levels. Blood lipid and glucose levels were measured using an Olympus^®^ AU 5400 automatic biochemical analyzer (Olympus Corporation, Japan, Shizuoka). Other indexes were estimated through conventional examination techniques.

#### 2.4.2 Diagnostic criteria for the MS

The diagnostic criteria for MS were referred to the International Diabetes Federation, the American Heart Association and the American Heart Association, Lung and Blood Institute in 2009 (22): ① obesity: BMI ≥ 25 kg/m^2^; ② hyperglycemia: FBG ≥ 5.6 mmol/L; ③ raised blood pressure: systolic blood pressure (SBP) ≥ 130 mmHg and (or) diastolic blood pressure (DBP) ≥ 85 mmHg; ④ TG ≥ 1.7 m mol/L; ⑤ HDL-C< 0.9 mmol/L for the males and <1.0 mmol/L for the female subjects. MS was diagnosed in three or more cases with the above risk factors.

#### 2.4.3 Lung cancer-associated autoantibody assay

1. Serum Specimen Collection: A total of 3 mL peripheral venous blood was collected from the subjects’ elbow vein after a 12 h fast. Blood was collected in a vacuum blood collection tube, which was then centrifuged at 3,000 × g for 10 min, after standing at room temperature for 30 min. The serum was separated and frozen at -80°C in a refrigerator.
2. Reagents and Instruments: The detection reagent was from a kit for the detection of seven types of autoantibodies =(Hangzhou Kaibaoluo Biotechnology Co., Ltd) was used. An ST-360 microplate reader from Shanghai Kehua Experimental System Co., Ltd. was employed.
3. Methods: Indirect enzyme-linked immunosorbent assay was performed to determine the concentrations of 7-AABs in serum samples. The detection process followed the procedures described in literature: Tumor-associated antigen (TAA) proteins were immobilized in microwells (50 mL/well) by a streptavidin tag. Serum samples diluted over [1∶109] with phosphate buffer were added to the microwells to bind the autoantibodies to the respective TAAs. Horseradish peroxidase-labeled antihuman IgG was added to each well to bind the autoantibodies. Serum autoantibody concentrations were measured using a standard curve of the serial dilution of positive antibodies (6).
4. Interpretation Method of Test Results: The 7-AABs detection kits provided positive cutoff values for each antibody. A positive result for a specific antibody was considered when a single-antibody detection value is higher than its corresponding cutoff value. If one or more antibodies showed positive results, the overall findings for the 7-AABs were considered positive (6).

### 2.5 Variables

In this study, the 7-AABs score was the dependent variable and the HDL-C level the independent variable. The covariates included age, BMI, SBP, DBP, FBG, and TG.

### 2.6 Statistical analysis

Statistical analysis was conducted using SPSS (version 27.0, SPSS, Chicago, IL, USA), Origin (version 2022b, OriginLab, USA), and R (version 4.3.3, The R Foundation) software. The normality of data was assessed via the Kolmogorov−Smirnov test. Continuous variables were presented as mean ± standard deviation (x ± s) or median M (Q1, Q3), depending on their distribution. Wilcoxon Mann−Whitney test and two-tailed Student’s t-test were used to analyze nonnormally and normally distributed data, respectively. A correlation heatmap among the five diagnostic indexes of MS, including serum HDL-C and the 7-AABs of lung cancer, was generated using Origin software. Multivariate logistic regression analysis was performed on the above indicators using R software, and a classification forest plot was created for visual representation. The restricted cubic spline logistic regression model was utilized to identify linear or nonlinear associations between the 7-AABs and HDL-C levels in the physical examination population. Objective calculations were performed to determine the cutoff value for the correlation between the 7-AABs concentration and HDL-C levels. A two-tailed *P <*0.05 was considered statistically significant.

## 3 Results

### 3.1 Participant characteristics

A total of 5,574 adult physical examination personnel, including 2,942 males and 2,632 females, were selected to participate in this study. Serum HDL-C levels were used to determine the characteristics of male and female participants, with different cutoffs for males (<0.9 and ≥0.9 mmol/L) and females (< 1.0 and ≥ 1.0 mmol/L) (22). As shown in **Table 1**, male participants with HDL-C levels < 0.9 mmol/L exhibited higher MS indicators (BMI, SBP, TG, and FBG), MS detection rates, and lung cancer autoantibody indicators (PGP9.5 and SOX2) compared with those in the HDL-C ≥ 0.9 mmol/L group. In the female cohort, participants with HDL-C levels < 1.0 mmol/L had higher age, MS indicators (BMI, SBP, TG, and FBG), and MS detection rates, No significant difference was observed in the 7-AABs between the two groups (**Table 2**).

**Table 1:** Characteristics of the study population.

Male (n=2942)				
HDL-C (mmol/L)	Total	Group1	Group2	*p*-Value
Age (year)	43 (33, 56)	43 (33,55)	43 (33,56)	0.899
BMI (kg/m2)	25.8 (23.8,28.1)	26.65 (24.325,29.4)	25.7 (23.7,28.0825)	<0.001***
DBP (mmHg)	78 (70,86)	79 (72,86)	78 (69,86)	0.062
SBP (mmHg)	132 (120,146)	148 (147,167)	130 (120,142)	<0.001***
FBG (mmol/l)	5.17 (4.77,5.81)	5.65 (5.1925,6.6075)	5.12 (4.75,5.7425)	<0.001***
TG (mmol/l)	1.722 (1.1975,2.5)	3.05 (1.9535,4.6575)	1.66 (1.16,2.37)	<0.001***
MS, n (%)				<0.001***
No	2241 (76.2)	55 (22.5)	2186 (81.0)	
Yes	701 (23.8)	189 (77.5)	512 (19.0)	
P53 (U/mL)	0.4 (0.1,1.0)	0.4 (0.1,1.3)	0.4 (0.1,1)	0.137
PGP9.5 (U/mL)	0.1 (0.1,0.4)	0.2 (0.1,0.8)	0.1 (0.1,0.4)	<0.001***
CAGE (U/mL)	0.1 (0.1,0.1)	0.1 (0.1,0.1)	0.1 (0.1,0.1)	0.844
SOX2 (U/mL)	0.5 (0.1,1.4)	0.7 (0.2,1.775)	0.5 (0.1,1.4)	0.035*
GAGE7 (U/mL)	0.7 (0.2,1.3)	0.8 (0.3,1.5)	0.6 (0.2,1.3)	0.060
GBU4-5 (U/mL)	0.3 (0.1,1.1)	0.4 (0.1,1.2)	0.3 (0.1,1.1)	0.300
MAGEA1 (U/mL)	0.1 (0.1,0.2)	0.1 (0.1,0.3)	0.1 (0.1,0.2)	0.053
7-AABs positive incidence, n (%)				0.586
No	2530 (86.0)	207 (84.8)	2323 (86.1)	
Yes	412 (14.0)	37 (15.2)	375 (13.9)	

HDL-C, high-density lipoprotein cholesterol; BMI, body mass index; DBP, diastolic blood pressure; SBP, systolic blood pressure; FBG, fasting blood glucose; TG, triglycerides; MS, Metabolic syndrome;p53, tumor protein 53; PGP9.5, protein gene product 9.5; CAGE, cancer/testis-associated antigen; SOX2, SRY-box containing gene 2; GAGE7, G antigen 7; GBU4-5, ATP-dependent RNA helicase; MAGE A1, melanoma antigen A1; 7 - AABs, 7-autoantibodies; n, number of subjects; %, weighted percentage *P < 0.05, ***P < 0.001.

**Table 2:** Characteristics of the study population.

Female (n=2632)				
HDL-C (mmol/L)	Total	Group1	Group2	*p*-Value
Age (year)	43 (33,57)	47 (34,58)	43 (32,56)	0.002**
BMI (kg/m2)	23.1 (21,25.6)	23.6 (21.2,26.4)	23 (20.96,25.5)	<0.001***
DBP (mmHg)	77 (69,85)	78 (70,85.5)	77 (68,85)	0.057
SBP (mmHg)	129 (117,142)	146 (131.5,150)	126 (116,139)	<0.001***
FBG (mmol/l)	4.95 (4.65,5.39)	5.2 (4.84,5.6)	4.92 (4.61,5.33)	<0.001***
TG (mmol/l)	1.6075 (1.13,2.34)	2.672 (1.7905,4.1)	1.48 (1.07,2.06)	<0.001***
MS, n (%)				<0.001***
No	2134 (81.1)	218 (51.3)	1916 (86.8)	
Yes	498 (18.9)	207 (48.7)	291 (13.2)	
P53 (U/mL)	0.4 (0.1,1)	0.4 (0.1,1)	0.4 (0.1,1)	0.856
PGP9.5 (U/mL)	0.1 (0.1,0.4)	0.1 (0.1,0.5)	0.1 (0.1,0.4)	0.426
CAGE (U/mL)	0.1 (0.1,0.1)	0.1 (0.1,0.1)	0.1 (0.1,0.1)	0.709
SOX2 (U/mL)	0.5 (0.1,1.3)	0.5 (0.1,1.45)	0.5 (0.1,1.3)	0.813
GAGE7 (U/mL)	0.7 (0.2,1.3)	0.7 (0.2,1.3)	0.6 (0.2,1.3)	0.797
GBU4-5 (U/mL)	0.3 (0.1,1.1)	0.3 (0.1,1.1)	0.3 (0.1,1.1)	0.914
MAGEA1 (U/mL)	0.1 (0.1,0.2)	0.1 (0.1,0.3)	0.1 (0.1,0.2)	0.340
7 - AABs positive incidence, n (%)				0.665
No	2308 (87.7)	370 (87.1)	1938 (87.8)	
Yes	324 (12.3)	55 (12.9)	269 (12.2)	

HDL-C, high-density lipoprotein cholesterol; BMI, body mass index;DBP, diastolic blood pressure; SBP, systolic blood pressure; FBG, fasting blood glucose; TG, triglycerides; MS, Metabolic syndrome;p53, tumor protein 53; PGP9.5, protein gene product 9.5;CAGE, cancer/testis-associated antigen; SOX2, SRY-box containing gene 2; GAGE7, G antigen 7; GBU4-5, ATP-dependent RNA helicase; MAGE A1, melanoma antigen A1; 7 - AABs, 7-autoantibodies; n, number of subjects; %, weighted percentage **P < 0.01, ***P < 0.001.

### 3.2 Association of MS-related indicators with 7-AABs concentration

**Figure 2:**
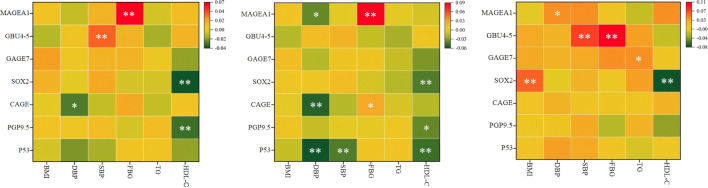
Pearson correlation analysis results of MS related indicators and 7- AABs. **(A)** for all, **(B)** for male, **(C)** for female. HDL-C, high-density lipoprotein cholesterol; BMI, body mass index; DBP, diastolic blood pressure; SBP, systolic blood pressure; FBG, fasting blood glucose; TG, triglycerides; MS, Metabolic syndrome;p53, tumor protein 53; PGP9.5, protein gene product 9.5;CAGE, cancer/testis‑associated antigen; SOX2, SRY‑box containing gene 2; GAGE7, G antigen 7; GBU4‑5, ATP‑dependent RNA helicase; MAGE A1, melanoma antigen A1; 7 - AABs, 7-autoantibodies; n, number of subjects; %, weighted percentage; *P < 0.05, **P < 0.01.

Pearson correlation analysis revealed the following trends: Among all participants, HDL-C was negatively correlated with PGP9.5 and SOX2. HDL-C was negatively correlated with P53, PGP9.5, and SOX2 in the male cohort and with SOX2 in the female cohort. Overall, HDL-C exhibited a significant negative correlation with SOX2, regardless of gender (*P* < 0.01) (**Figure 2**).

### 3.3 Association of MS-related indicators with combined test results on the 7-AABs

**Figure 3:**
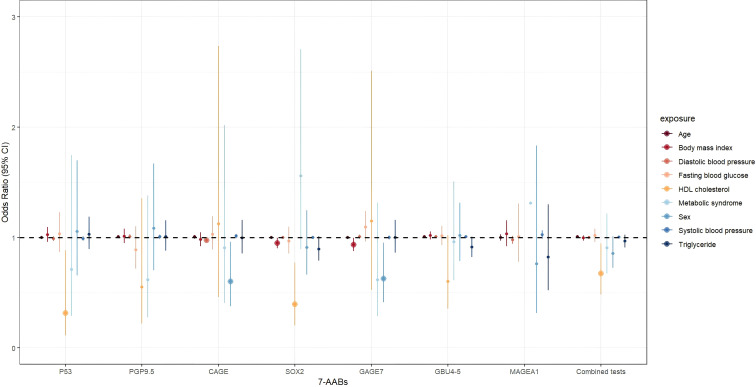
The association between MS and general indicators and 7-AABs combined test results in all participants. exposure: Age; Sex; BMI, body mass index; DBP, diastolic blood pressure; SBP, systolic blood pressure; FBG, fasting blood glucose; TG, triglycerides; HDL-C, high-density lipoprotein cholesterol; MS, Metabolic syndrome.

After the adjustment for age, sex, BMI, and various MS indicators, such as DBP, SBP, FBG, and TG, the research demonstrated a significant decrease in the risk of positive results for the combined 7-AABs test with the increase in the HDL-C levels (OR = 0.675, 95% confidence interval (CI): 0.483–0.944, *P* = 0.022). Similarly, the risk of positive P53 and SOX2 test results significantly decreased with increased HDL-C levels (OR = 0.314, 95% CI: 0.111–0.885, *P* = 0.028; OR = 0.396, 95% CI: 0.203–0.772, *P* = 0.007). However, HDL-C showed no effect of on the detection of PGP9.5 (OR = 0.549, 95% CI: 0.222–1.356, *P* = 0.194), CAGE (OR = 1.123, 95% CI: 0.461–2.734, *P* = 0.799), GAGE7 (OR = 1.149, 95% CI: 0.526–2.509, *P* = 0.728), GBU4-5 (OR = 0.600, 95% CI: 0.353–1.020, *P* = 0.059), or MAGEA1 (OR = 4.126, 95% CI: 0.958–17.762, *P* = 0.057). **Table 3** and **Figure 3** provide additional details.

**Table 3:** Relationship between MS indicators and 7- AABs test results.

	P53		PGP9.5		CAGE		SOX2	
	β (95% CI)	*P* value	β (95% CI)	*P* value	β (95% CI)	*P* value	β (95% CI)	*P* value
Sex	1.054 (0.654,1.697)	0.83	1.082 (0.701,1.67)	0.722	0.602 (0.377,0.961)	0.034	0.909 (0.663,1.246)	0.552
Age	0.999 (0.984,1.015)	0.919	1.008 (0.994),1.022	0.288	1.005 (0.99,1.02)	0.533	1.001 (0.991,1.012)	0.839
BMI	1.024 (0.96,1.093)	0.463	1.012 (0.949,1.08)	0.71	0.981 (0.919,1.048)	0.572	0.948 (0.903,0.995)	0.03
DBP	0.989 (0.968,1.011)	0.32	1.007 (0.988,1.027)	0.458	0.975 (0.956,0.995)	0.014	0.998 (0.985,1.012)	0.809
SBP	0.99 (0.969,1.011)	0.338	1.005 (0.985,1.024)	0.643	1.013 (0.993,1.033)	0.195	1.001 (0.987,1.015)	0.872
FBG	1.033 (0.869,1.227)	0.712	0.888 (0.718,1.099)	0.274	1.03 (0.89,1.192)	0.695	0.968 (0.854,1.098)	0.616
TG	1.03 (0.893,1.189)	0.685	1.007 (0.879,1.154)	0.919	0.995 (0.855,1.158)	0.948	0.893 (0.788,1.011)	0.074
HDL-C	0.314 (0.111,0.885)	0.028	0.549 (0.222,1.356)	0.194	1.123 (0.461,2.734)	0.799	0.396 (0.203,0.772)	0.007
MS	0.71 (0.288,1.746)	0.455	0.617 (0.276,1.381)	0.24	0.904 (0.404,2.019)	0.805	1.557 (0.896,2.706)	0.117
	GAGE7		GBU4-5		MAGEA1		Combined tests	
	β (95% CI)	*P* value	β (95% CI)	*P* value	β (95% CI)	*P* value	β (95% CI)	*P* value
Sex	0.627 (0.413,0.952)	0.028	1.016 (0.786,1.314)	0.902	0.761 (0.316,1.831)	0.543	0.855 (0.725,1.01)	0.065
Age	1 (0.986,1.013)	0.974	1.006 (0.997,1.014)	0.173	0.999 (0.971,1.027)	0.927	1.005 (0.999,1.01)	0.095
BMI	0.934 (0.877,0.994)	0.031	1.017 (0.981,1.053)	0.356	1.031 (0.92,1.154)	0.6	0.994 (0.97,1.018)	0.617
DBP	1.008 (0.99,1.026)	0.4	1.005 (0.994,1.016)	0.375	0.976 (0.941,1.013)	0.199	1 (0.993,1.007)	0.978
SBP	1 (0.982,1.019)	0.971	1.005 (0.994,1.017)	0.376	1.025 (0.988,1.063)	0.193	1.002 (0.995,1.009)	0.598
FBG	1.092 (0.962,1.239)	0.173	1.013 (0.93,1.103)	0.772	1.008 (0.778,1.307)	0.951	1.018 (0.961,1.078)	0.55
TG	0.999 (0.861,1.159)	0.991	0.911 (0.823,1.008)	0.07	0.823 (0.522,1.299)	0.403	0.965 (0.91,1.023)	0.227
HDL-C	1.149 (0.526,2.509)	0.728	0.6 (0.353,1.02)	0.059	4.126 (0.958,17.762)	0.057	0.675 (0.483,0.944)	0.022
MS	0.614 (0.287,1.314)	0.209	0.959 (0.61,1.507)	0.856	1.312 (0.296,5.819)	0.721	0.905 (0.673,1.216)	0.507

BMI, body mass index; DBP, diastolic blood pressure; SBP, systolic blood pressure; FBG, fasting blood glucose; TG, triglycerides; HDL-C, high-density lipoprotein cholesterol; MS, Metabolic syndrome.

### 3.4 Association between the serum HDL-C level and the odds of testing positive for the 7-AABs

**Figure 4:**
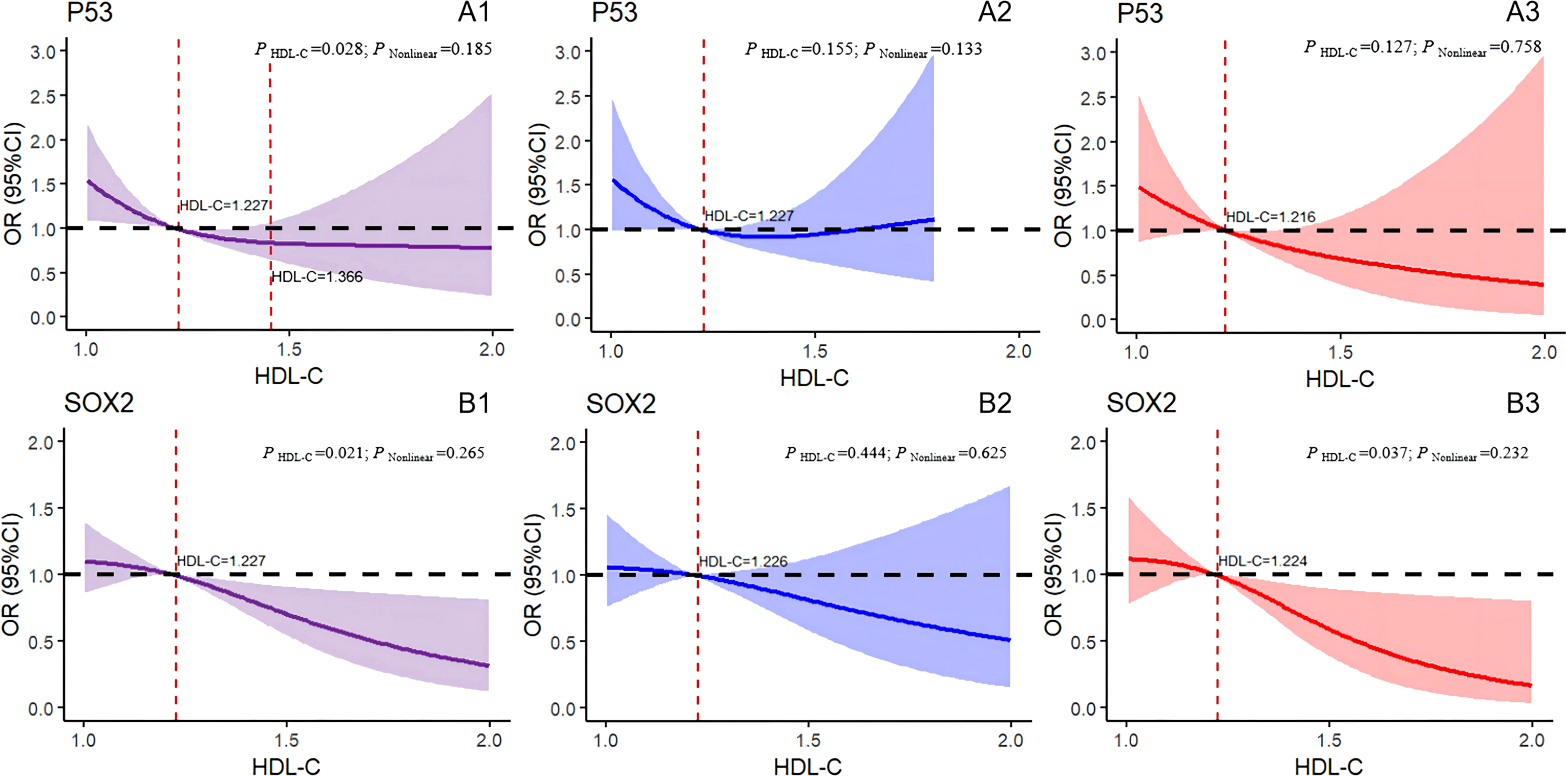
Logistic regression models with cubic natural spline analysis of HDL-C on a continuous scale and odds of positive 7-AABs. **(A1–A3)** for P53, **(B1–B3)** for SOX2. Multivariate regression models, adjusted for age, BMI, DBP, SBP, FBG,TG, and MS. Odds ratios are indicated by solid lines, and 95% CIs are represented by shaded areas (the shaded area located above the black dashed line refers to the promotion of positive risk occurrence; that located below the black dotted line denotes the obstructed positive risk occurrence; shaded areas spanning the black dashed line mean no statistical significance). *P*_Nonlinear_, P for nonlinearity. Purple for all participants; blue for all males; red for all females. P53, tumor protein 53; SOX2, SRY-box containing gene 2; HDL-C, high-density lipoprotein cholesterol.

**Figure 5:**
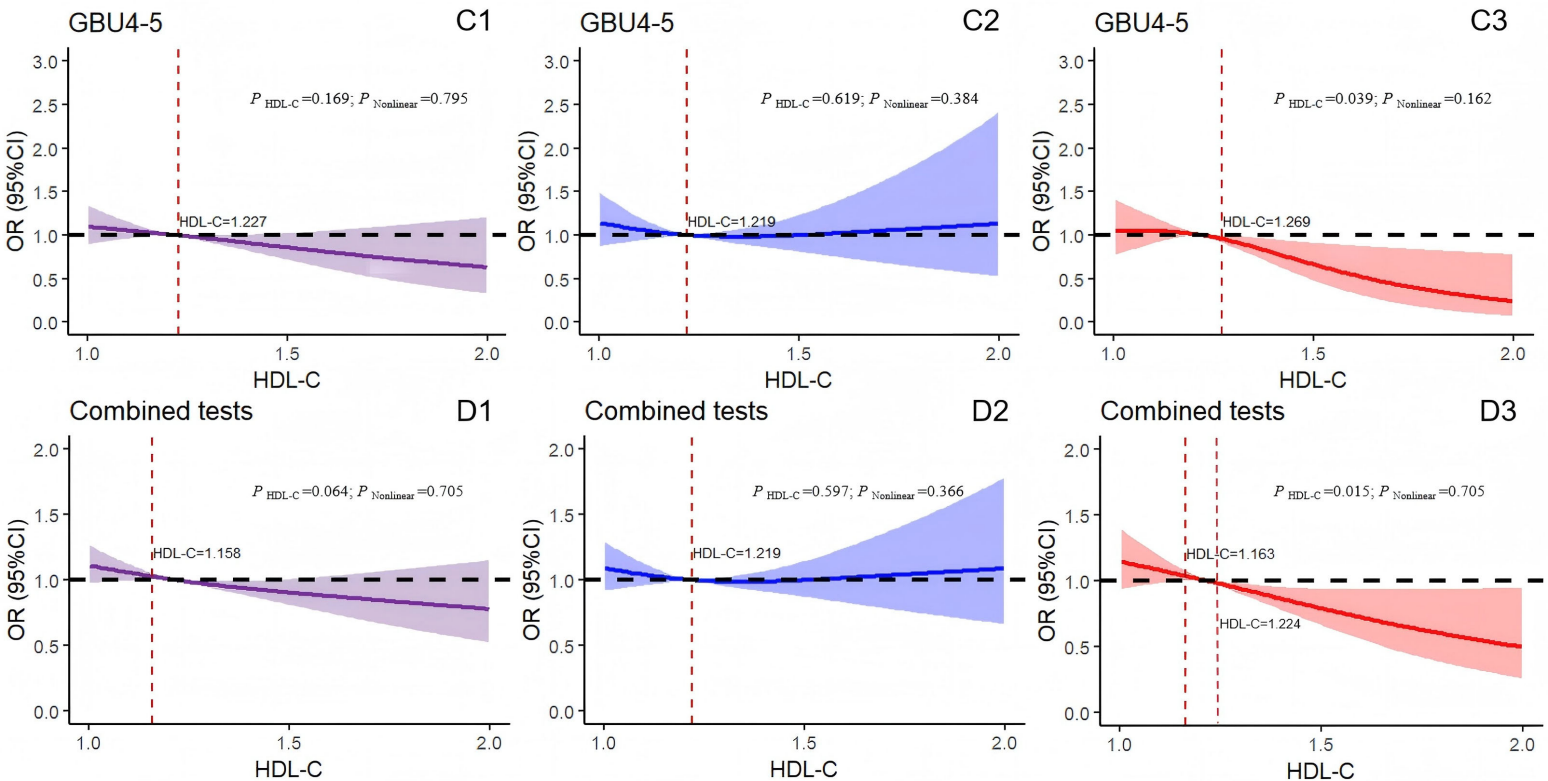
Logistic regression models with cubic natural spline analysis of HDL-C on a continuous scale and odds of positive 7-AABs. **(C1–C3)** for GBU4-5 and **(D1–D3)** for 7-AABs combined tests. Multivariate regression models adjusted for age, BMI, DBP, SBP, FBG, TG, and MS. Odds ratios are indicated by solid lines and 95% CIs by shaded areas (the shaded area located above the black dashed line represents the promotion of positive risk occurrence; that located below the black dotted line denotes the obstructed positive risk occurrence; shaded areas spanning the black dashed line indicate no statistical significance). *P*_Nonlinear_, *P* for nonlinearity. Purple for all participants; blue for all males; red for all females. GBU4-5, ATP-dependent RNA helicase; combined tests: 7-AABs combined tests; HDL-C, high-density lipoprotein cholesterol.

To present the analysis findings in a more visual manner, we conducted restricted cubic spline regression, and the results are displayed in **Figures 4** and **5**. After the adjustment for age, BMI, DBP, SBP, FBG, TG, and MS, the overall results showed a significant linear association between the hazard of positive P53 detection and HDL-C levels (*P*_HDL-C_=0.028, *P*_Nonlinear_=0.185). Specifically, the HDL-C levels between 0 and 1.227 mmol/L correlated with an increased risk of positive P53 in the tests. However, the HDL-C levels between 1.227 and 1.366 mmol/L correlated with a decreased risk of positive P53 (**Figure 4A1**). No linear nor nonlinear associations were found between HDL-C and P53 levels in the male (*P*_HDL-C_ = 0.155, *P*_Nonlinear_ = 0.133; **Figure 4A2**) and female (*P*_HDL-C_ = 0.127, *P*_Nonlinear_ = 0.758; **Figure 4A3**) cohorts. **Figure 4B1** confirms a significant linear association between HDL-C levels and the hazard of positive SOX2 test results (*P*_HDL-C_ = 0.021, *P*_Nonlinear_ = 0.265). Specifically, at the serum HDL-C level greater than 1.227 mmol/L, the risk of positive SOX2 test results decreased with the increase in HDL-C levels. However, no association was found between the HDL-C levels and SOX2 test results in the male (*P*_HDL-C_ = 0.444, *P*_Nonlinear_ = 0.625; **Figure 4B2**) and female (*P*_HDL-C_ = 0.037, *P*_Nonlinear_ = 0.232; **Figure 4B3**) cohorts.

**Figure 5C1** indicates the absence of linear or nonlinear associations between the results of the GBU 4-5 test and HDL-C levels (*P*_HDL-C_ = 0.169, *P*_Nonlinear_ = 0.795). Similarly, the GBU 4-5 test results and HDL-C levels in the male group showed no association (*P*_HDL-C_ = 0.619, *P*_Nonlinear_ = 0.384; **Figure 5C2**). By contrast, in the female group, when HDL-C exceeded 1.269 mmol/L, the risk of positive GBU 4-5 test results decreased with the increase in HDL-C levels (*P*_HDL-C_ = 0.039, *P*_Nonlinear_ = 0.162; **Figure 5C3**). Furthermore, the concentration of HDL-C in the total participants displayed no correlation with the 7-AABs joint detection results (*P*_Nonlinear_ = 0.705, r = 0.064) (**Figure 5D1**). Similarly, in the male group (**Figure 5D2**), no linear nor nonlinear correlation was observed between the HDL-C levels and 7-AABs combined detection results (*P*_HDL-C_ = 0.597, *P*_Nonlinear_ = 0.366). By contrast, in the female group (**Figure 5D3**), a significant association was detected between the HDL-C levels and 7-AABs combined detection results (*P*_HDL-C_ = 0.015, *P*_Nonlinear_ = 0.705). Specifically, the HDL-C levels between 1.163 and 1.224 mmol/L correlated with an increased risk of positive 7-AABs test results. However, when the concentration of HDL-C exceeded 1.224 mmol/L, the risk of positive 7-AABs combined test results decreased with the increase in HDL-C levels. In addition, no linear nor nonlinear associations were recorded between the serum HDL-C levels and detection results for PGP9.5, CAGE, GAGE7, GBU 4-5, or MAGEA1.

## 4 Discussion

This research aimed to explore the correlation between the serum HDL-C and detection results of 7-AABs in a physical examination population. Highly homogeneous samples (n = 5,574) aged ≥ 18 years and who underwent physical examination within six years were included in this study. After the adjustment for variables, such as age, BMI, etc., certain concentrations of HDL-C were associated with a reduced risk of positive test results for P53, SOX2, GBU 4-5, and the 7-AABs. Notably, in women, the HDL-C levels between 1.163 and 1.224 mmol/L correlated with an increased risk of positive 7-AABs test results. However, this phenomenon was not observed in the male group. Thus, this research provides evidence of a sex-specific association between the 7-AABs and serum HDL-C in a Chinese physical examination population, independent of age, BMI, etc.

Metabolic reprogramming is an important indicator of cancer (23). Lipid metabolism plays a crucial role in this process because the resulting signaling molecules can modify the TME and stimulate tumor cell proliferation, invasion, and metastasis (24). In addition, HDL-C can regulate antitumor immunity (25), promote T cell aggregation, and increase the influx of macrophages into the TME (26). Lung cancer, which is responsible for the majority of cancerrelated deaths worldwide, is often diagnosed at advanced stages, when it is incurable (5). Current biological and epidemiological evidence increasingly supports the idea that reduced levels of HDL-C contribute to a high risk of lung cancer (14, 17). Multiple molecular abnormalities can be detected throughout tumor development (27). The immune system of lung cancer patients releases specific autoantibodies against TAAs through the production of high-affinity T cells, which can serve as biomarkers. These autoantibodies are more stable than the antigens themselves; they exhibit higher levels and remain detectable in the serum for many years (28). Furthermore, these autoantibodies can be detected during the asymptomatic phase of lung cancer (29). However, the fundamental association and interaction between serum HDL-C and the 7-AABs remain unexplored domestically and internationally.

In this study, prior to adjustment for age, BMI, and metabolism-related confounding factors, serum HDL-C showed a negative correlation with PGP9.5 and SOX2 in all participants. HDL-C was negatively correlated with P53, PGP9.5, and SOX2 in men and negatively correlated with SOX2 in women. PGP9.5 is a ubiquitin hydrolase found in neurons and neuroendocrine cells. This enzyme can be abnormally expressed and secreted in lung cancer cells, which leads to humoral reactions. Elevated PGP9.5 expression is associated with lung cancer development and is prevalent in lung cancer cell lines (30). The P53 gene is one of the earliest discovered anti-oncogenes (31). During its mutation under the influence of various factors, the resulting P53 protein promotes tumor cell survival, inhibits apoptosis, and leads to cancer development (32). This mutation also induces the production of p53 antibodies in the blood (33). Mutant p53 is correlated with a high incidence of cell-in-cell structures in lung adenocarcinoma, which is a common feature and a crucial predictor of poor prognosis and disease recurrence (34). SOX2 is a transcription factor associated with sex-determining genes. This factor possesses an HMG-specific domain. SOX2 is often overexpressed in squamous cell carcinoma and small-cell lung cancer (35). Furthermore, it serves as an independent predictor of poor prognosis in lung adenocarcinoma (36). This study revealed that serum HDL-C concentration was inversely associated with the levels of PGP9.5, P53, SOX2, and other autoantibodies. The underlying mechanism may involve immune regulation and the capability of HDL-C to suppress inflammation (37) and its inhibition of tumor cell proliferation or growth (38).

In regard to the association between malignant tumor hazards and HDL-C levels, extensive research has consistently shown an inverse correlation between HDL-C and cancer hazards (39). Dessi et al. revealed significantly lower levels of HDL-C in a lung cancer cohort compared with a healthy control group (40). After adjusting for confounding factors, such as age, BMI, DBP, SBP, FBG, TG, and MS, this research also demonstrated a distinctly negative correlation between the detection of 7-AABs and HDL-C in the female cohort. The risk of positive test results for SOX2, GBU4-5, and 7-AABs gradually decreased when the serum HDL-C levels increased to ≥1.2 mmol/L. Numerous studies have proven the connection between cancer occurrence and development and chronic inflammation. Low levels of serum HDL-C may indicate a reduced ability to resist inflammation and consequently promote the advancement of lung cancer (41). The results of this study suggest that increased serum HDL-C levels may contribute to tumor suppression through the enhancement of the anti-inflammatory properties of HDL-C (42). This reduction in the body’s inflammatory response can potentially delay the progression of lung cancer and subsequently decrease the levels of autoantibodies. However, this study did not observe any effect of serum HDL-C on any autoantibody test results in the cohort of men. According to Luo et al., P53, PGP 9.5, SOX 2, GAGE 7, and GBU 4-5 levels do not differ in gender (35). The reason may be that differences in sexual habits and hormone secretion between men and women affect the level of HDL-C. Usually, the normal range of HDL-C is higher in women than in men. Moreover, serum HDL-C is speculated to be more sensitive to the early monitoring of lung cancer autoantibodies in women than in men.

However, the increase in serum HDL-C levels do not necessarily equate to improved outcomes. Zhong et al. reported that the lowest hazard of malignant tumor-related death was associated with HDL-C levels between 64–68 mg/dL (12). The lowest hazard was observed at 56–66 mg/dL in males and at 66–73 mg/dL in females. Compared with the HDL-C level of 66 mg/dL, each increase or decrease of 10 mg/dL in the HDL-C level was associated with risk ratios of cancer death of 1.02 and 1.11, respectively. Furthermore, some Mendelian randomized studies have failed to establish a definitive association between cancer hazards, including those of lung cancer, and HDL-C (43, 44). By contrast, one research suggested that high HDL-C levels were associated with an increased risk of breast cancer (44). The present research also revealed a similar phenomenon. Among all participants, the HDL-C levels between 0 and 1.227 mmol/L correlated with an increased risk of positive P53 test findings. However, when the HDL-C was ≥ 1.366 mmol/L, no difference was observed in P53 detection. In the female cohort, HDL-C levels between 1.163 and 1.224 mmol/L correlated with an increased risk of positive 7-AABs in the tests. Moreover, this study failed to reveal any association among CAGE, GAGE 7, MAGEA1, and HDL-C with or without adjusting for confounding factors, such as age and sex. These antibodies are speculated to be less sensitive as those such as P53, which may indicate a high false negative rate in some lung cancer-related antibody profiles (45) and may be related to whether marital status, socioeconomic factors, or dietary habits were excluded in the study; further research is still needed in the future. This work is the first to identify the varying effects of serum HDL-C levels on lung cancer autoantibodies at different levels. Therefore, it holds immense significance and has the potential to establish HDL-C as an early monitoring indicator for lung cancer. The combined detection of 7-AABs and HDL-C (HDL-C≥1.224 mmol/L, *P* = 0.015) may improve the sensitivity and specificity of lung cancer screening, and its diagnostic efficacy is expected to be higher than that of pure antibody detection.

## 5 Study strengths and limitations

The study participants were carefully selected to ensure the applicability of the findings to a broader population. In addition, through the utilization of advanced statistical analysis methods, such as multivariate logistic regression analysis and restricted cubic spline logistic regression model, this study revealed a detailed dose–response association between serum HDL-C and 7-AABs. The study’s robustness and interpretability were enhanced by adjusting for covariates, such as age. However, this research has limitations. First, as a cross-sectional study, the establishment of a causal association between serum HDL-C and 7-AABs levels was challenging, and the effect of HDL-C on 7-AABs requires further validation via longitudinal studies. Second, this research disregarded certain demographic indicators, such as marital status, occupation, and educational level. Lastly, the data used were all obtained from the same physical examination department, which limits the generalizability of the results. In the future, a multicenter follow-up study involving multiple hospitals will be conducted to verify the generalizability of the findings of this work. Therefore, the conclusions of this research must be validated through comparative analysis in the context of multiple healthcare institutions.

## 6 Conclusion

The findings strongly support the complex correlations between serum HDL-C and 7-AABs. The results indicate that high levels of HDL-C are highly associated with a reduced risk of positive 7-AABs detection, which suggests that HDL-C holds potential as an effective biomarker for the assessment of an individual’s cancer risk. Based on these observations, future research should include prospective cohort studies to further validate the feasibility of using HDL-C as a predictive bioindicator for cancer potential to provide a scientific basis for the early prevention and treatment of cancer.

## Data Availability

The original contributions presented in the study are included in the article/supplementary material. Further inquiries can be directed to the corresponding author.
